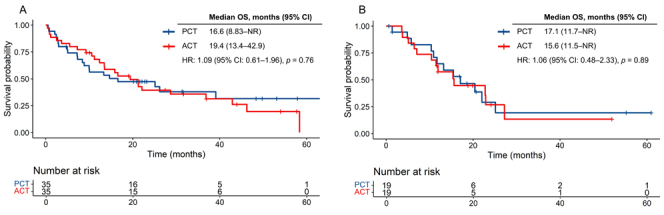# A Multicenter, Retrospective, Real-World Study of Atezolizumab Plus Chemotherapy and Pembrolizumab Plus Chemotherapy for Older Patients With NSCLC

**DOI:** 10.1016/j.jtocrr.2025.100891

**Published:** 2025-08-21

**Authors:** Kensuke Kanaoka, Kinnosuke Matsumoto, Takayuki Shiroyama, Akihiro Tsukaguchi, Nao Shoshihara, Koki Moritomo, Yuhei Kinehara, Yasuhiro Mihashi, Tomoki Kuge, Midori Yoneda, Soichiro Kato, Keijiro Yamauchi, Hirotomo Machiyama, Yuki Nishikawa, Osamu Morimura, Akito Miyazaki, Kiyohide Komuta, Kouji Azuma, Satoshi Tanaka, Toshie Niki, Akio Osa, Akihiro Tamiya, Motohiro Tamiya, Hidekazu Suzuki, Masahide Mori, Yoshito Takeda, Atsushi Kumanogoh

**Affiliations:** a Department of Respiratory Medicine and Clinical Immunology, Graduate School of Medicine, The University of Osaka, Japan; b Department of Internal Medicine, National Hospital Organization Kinki-Chuo Chest Medical Center, Osaka, Japan; c Department of Respiratory Medicine, Osaka Keisatsu Hospital, Osaka, Japan; d Department of Respiratory Medicine and Clinical Immunology, Nippon Life Hospital, Osaka, Japan; e Department of Thoracic Oncology, National Hospital Organization Osaka Toneyama Medical Center, Osaka, Japan; f Department of Respiratory Medicine, Ikeda Municipal Hospital, Osaka, Japan; g Department of Thoracic Oncology, Osaka Habikino Medical Center, Osaka, Japan; h Department of Immunogenomic Medicine, Research Institute, National Cancer Center, Tokyo, Japan; i Department of Respiratory Medicine, Toyonaka Municipal Hospital, Osaka, Japan; j Department of Respiratory Medicine, Osaka International Cancer Institute, Osaka, Japan; k Department of Respiratory Medicine, National Hospital Organization Osaka National Hospital, Osaka, Japan; l Local Independent Administrative Agency Osaka Prefectural Hospital Organization Osaka General Medical Center, Osaka, Japan; m Department of Respiratory Medicine, Nishinomiya Municipal Central Hospital, Hyogo, Japan; n Department of Respiratory Medicine, Kinki Central Hospital, Hyogo, Japan; o Department of Immunopathology, Immunology Frontier Research Center, The University of Osaka, Japan; p Center for Advanced Modalities and DDS, The University of Osaka, Japan; q Center for Infectious Diseases for Education and Research, The University of Osaka, Japan; r Integrated Frontier Research for Medical Science Division, Institute for Open and Transdisciplinary Research Initiatives, The University of Osaka, Japan; s Japan Agency for Medical Research and Development-Core Research for Evolutional Science and Technology, The University of Osaka, Japan

**Keywords:** Atezolizumab, Immune checkpoint inhibitors, Immune-related adverse events, Non–small cell lung cancer, Pembrolizumab, Pneumonitis

## Abstract

**Introduction:**

Evidence of immune checkpoint inhibitors (ICIs) combined with chemotherapy for older patients with NSCLC is limited. This real-world study compared the efficacy and safety of atezolizumab plus chemotherapy (ACT) with those of pembrolizumab plus chemotherapy (PCT) for older patients with advanced nonsquamous NSCLC.

**Methods:**

This multicenter, retrospective study included 288 patients 65 years or older with advanced or recurrent nonsquamous NSCLC who received PCT or ACT as first-line treatment at 13 institutions in Japan. After one-to-one propensity score matching, overall survival (OS), the incidence of grade 3 or higher treatment-related adverse events, and all-grade pneumonitis of the PCT and ACT groups were compared.

**Results:**

After propensity score matching, 54 patients were included in each of the groups. OS did not significantly differ between the PCT and ACT groups. The median OS was 16.6 months for both groups. Compared with the PCT group, the ACT group had a hazard ratio of 1.09 (95% confidence interval [CI]: 0.68–1.74; *p* = 0.7). Grade 3 or higher adverse events occurred in 40.7% and 33.3% of patients in the PCT and ACT groups, respectively (*p* = 0.55). The incidence of treatment-related pneumonitis of the PCT group was significantly higher (29.6%, including 11 grade ≥3 cases) than that of the ACT group (5.6%, including two grade ≥3 cases) (*p* = 0.002).

**Conclusions:**

ACT may be associated with a more favorable safety profile than that of PCT for the Japanese population; therefore, ACT could be considered a treatment option for older patients with advanced nonsquamous NSCLC.

## Introduction

Lung cancer accounts for an estimated 1.8 million deaths worldwide and is the leading cause of cancer-related deaths.^1^ Although more than half of all patients with lung cancer are diagnosed after age 65 years, older patients are often underrepresented in clinical trials.^2^^,^^3^ Therefore, evidence that supports therapeutic strategies for this population is often derived from real-world data or subgroup analyses of clinical trials. Although the efficacy and safety of certain chemotherapy regimens and immune checkpoint inhibitor (ICI) monotherapies have been exhibited, clear recommendations for older patients with NSCLC without driver mutations have not yet been established.^3^, ^4^, ^5^, ^6^, ^7^, ^8^

Evidence regarding the combination of ICIs and chemotherapy is limited, and such regimens have not yet been widely recommended for older patients.^9^ The KEYNOTE-189 study reported the efficacy of pembrolizumab plus chemotherapy (PCT) for patients 65 years or older as well as those younger than 65 years.^10^ A real-world study that compared ICI monotherapy plus chemotherapy for patients 75 years or older found that no survival benefit resulted from the addition of chemotherapy and reported a higher incidence of grade 3 or higher (grade ≥3) immune-related adverse events (AEs).^11^ Meanwhile, an integrated analysis of the IMpower130 and IMpower132 trials revealed that atezolizumab plus chemotherapy (ACT) improved overall survival (OS) when compared with chemotherapy alone for a subgroup of patients 75 years or older.^12^ However, no real-world studies have specifically evaluated the efficacy and safety of ACT for older patients; furthermore, to date, no studies have directly compared ACT and PCT. Therefore, the present study aimed to compare the efficacy and safety of ACT with those of PCT for older patients with advanced or recurrent nonsquamous NSCLC.

## Material and Methods

### Study Design and Patients

We conducted a multicenter, retrospective cohort study of patients from 13 institutions in Japan. Eligible patients were 65 years or older who had cytologically or histologically confirmed advanced or recurrent nonsquamous NSCLC and received first-line treatment with one of the following regimens between December 2018 and December 2022: atezolizumab plus carboplatin plus nanoparticle albumin-bound paclitaxel (nab-PTX); atezolizumab plus carboplatin plus pemetrexed; pembrolizumab plus carboplatin plus pemetrexed; or pembrolizumab plus cisplatin plus pemetrexed. Patients with major *EGFR* gene mutations or *ALK* fusion gene rearrangements were excluded. This retrospective cohort study was conducted in accordance with the Declaration of Helsinki and the WHO’s guidelines for Good Clinical Practice. Ethical approval was obtained from the institutional review boards of all participating institutions. An opt-out approach was used; thus, providing the patients and their families with the opportunity to decline participation.

### Data Collection

Clinical data extracted from medical records included age, sex, Eastern Cooperative Oncology Group performance status, smoking status, preexisting interstitial lung disease (ILD), clinical stage, histologic diagnosis, programmed cell death ligand 1 (PD-L1) expression, *EGFR* mutations, *ALK* rearrangements, treatment regimens, clinical outcomes, and treatment-related AEs. Treatment efficacy was evaluated according to the Response Evaluation Criteria in Solid Tumors version 1.1. Tumor response was assessed by clinicians and categorized as a complete response, partial response, stable disease, or progressive disease. OS was defined as the time from the initiation of first-line treatment to the date of death from any cause. Data regarding grade ≥3 AEs and all-grade pneumonitis were collected. The incidence and severity of AEs were determined by clinicians according to the Common Terminology Criteria for Adverse Events version 5.0.

### Statistical Analysis

The primary end point was OS of patients treated with either PCT or ACT after propensity score matching (PSM). Secondary end points included grade ≥3 AEs and all-grade pneumonitis. An age subgroup analysis was performed as an exploratory analysis. PSM was performed using the propensity scores of the patients; these scores were calculated using logistic regression with age, sex, smoking status, Eastern Cooperative Oncology Group performance status, preexisting ILD, stage, histologic diagnosis, PD-L1 expression, and metastases to the brain, liver, and pleura as covariates. We performed one-to-one PSM using the nearest neighbor method with a caliper width of 0.2. Covariate balance between the matched groups was assessed using the standardized mean difference (SMD). An SMD less than 0.2 indicated acceptable balance. Patient characteristics are described as medians and interquartile ranges for quantitative variables and as counts and percentages for qualitative variables. We conducted the chi-square test (Fisher’s exact tests for small numbers) to compare categorical variables and the Wilcoxon rank-sum test to compare continuous variables before and after PSM. The OS was estimated using a Kaplan-Meier analysis and compared using the log-rank test. Hazard ratios (HRs) for OS were calculated using the Cox proportional hazards regression model. A two-sided *p* less than 0.05 was considered statistically significant. All statistical analyses were performed using R software (version 4.4.2; R Foundation for Statistical Computing, Vienna, Austria) and RStudio (version 2024.12.0+467; Posit PBC, Boston, MA).

## Results

### Patient Flow and Baseline Characteristics

**Supplementary Figure 1:**
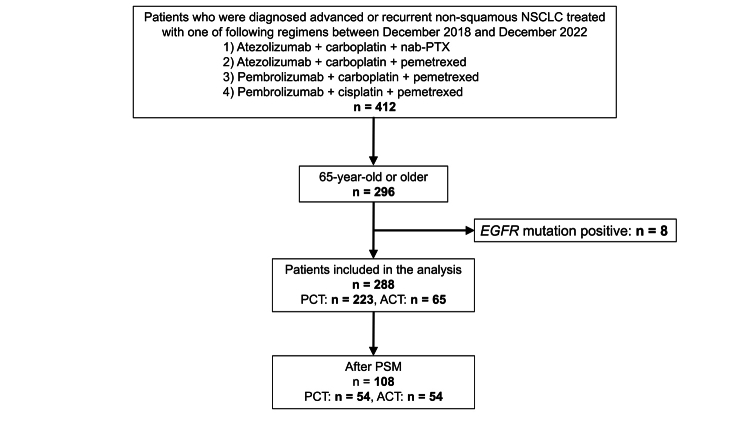


During the study period, 412 patients received one of the aforementioned regimens specified for this study to treat advanced or recurrent nonsquamous NSCLC. Among them, 296 patients were 65 years or older. Eight patients with *EGFR* mutations were excluded, and no patients with *ALK* rearrangements were identified in either group; therefore, 288 patients were included in the final analysis. Among these patients, 223 received PCT and 65 received ACT. After PSM, 54 patients and 54 patients were included in the PCT and ACT groups, respectively. The SMDs for all covariates were less than 0.2, indicating that adequate balance was achieved between the matched groups. A CONSORT diagram illustrating patient selection is shown in Supplementary Figure 1.

Baseline characteristics of the 288 patients before PSM and those of the 108 patients after PSM are presented in Table 1. After PSM, baseline characteristics of the PCT and ACT groups were not significantly different. The ACT group included 34 patients who received carboplatin plus nab-PTX and 20 patients who received carboplatin plus pemetrexed as concurrent chemotherapy. In the PCT group, all patients received platinum plus pemetrexed and three patients received cisplatin.

**Table 1:** Baseline Patient Characteristics Before and After Propensity Score Matching

Characteristics	Before PSM			After PSM			
	PCT (n = 223)	ACT (n = 65)	*p* Value	PCT (n=54)	ACT (n=54)	*P* Value	SMD
Age (median [IQR])	72 [69–76]	73 [71–77]	0.23	73 [70–76.8]	72 [70–76]	0.57	0.14
Sex (%)			0.82				0.13
Female	53 (23.7)	17 (26.2)		15 (27.8)	12 (22.2)		
Male	170 (76.2)	48 (73.8)		39 (72.2)	42 (77.8)	0.66	
ECOG performance status (%)			0.27			0.71	
0	71 (31.8)	16 (24.6)		10 (18.5)	14 (25.9)		0.17
1	129 (57.8)	46 (70.8)		40 (74.1)	37 (68.5)		0.12
2	21 (9.4)	3 (4.6)		4 (7.4)	3 (5.6)		0.09
3	2 (0.9)	0 (0.0)		0 (0.0)	0 (0.0)		
Smoking history (%)			0.56				0.11
Never	40 (17.9)	9 (13.8)		9 (16.7)	7 (13.0)	0.79	
Current or former	183 (82.1)	56 (86.2)		45 (83.3)	47 (87.0)		
Stage (%)			0.52			0.86	
III	17 (7.6)	5 (7.7)		5 (9.3)	5 (9.3)		0.0
IVA	79 (35.4)	27 (41.5)		24 (44.4)	24 (44.4)		0.0
IVB	99 (44.4)	29 (44.6)		23 (42.6)	21 (38.9)		0.07
Recurrence	28 (12.6)	4 (6.2)		2 (3.7)	4 (7.4)		0.15
histology (%)			<0.001			>0.99	
Ad	199 (89.2)	46 (70.8)		46 (85.2)	46 (85.2)		0.0
NOS	21 (9.4)	13 (20.0)		8 (14.8)	8 (14.8)		0.0
other	3 (1.3)	6 (9.2)		0 (0.0)	0 (0.0)		0.0
PD-L1 tumor proportion score, %			0.56			0.69	
≥50%	61 (27.4)	17 (26.2)		10 (18.5)	14 (25.9)		0.16
1–49%	50 (22.4)	20 (30.8)		14 (25.9)	16 (29.6)		0.08
<1%	73 (32.7)	19 (29.2)		21 (38.9)	17 (31.5)		0.16
unknown	39 (17.5)	9 (13.8)		9 (16.7)	7 (13.0)		0.11
Preexisting ILD (%)	25 (11.2)	11 (16.9)	0.31	6 (11.1)	7 (13.0)	>0.99	0.05
Brain metastases (%)			0.27			>0.99	0.0
negative	180 (80.7)	57 (87.7)		47 (87.0)	47 (87.0)		
positive	43 (19.3)	8 (12.3)		7 (13.0)	7 (13.0)		
Liver metastases (%)			0.87			>0.99	
negative	197 (88.3)	57 (87.7)		48 (88.9)	49 (90.7)		0.06
positive	25 (11.2)	8 (12.3)		6 (11.1)	5 (9.3)		0.06
unknown	1 (0.4)	0 (0.0)		0 (0.0)	0 (0.0)		0.0
Pleural metastases (%)			0.54			0.85	0.08
negative	129 (57.8)	41 (66.1)		31 (57.4)	33 (61.1)		
positive	94 (42.2)	24 (36.9)		23 (42.6)	21 (38.9)		

ACT, Atezolizumab plus chemotherapy; Ad, adenocarcinoma; ECOG, Eastern Cooperative Oncology Group; ILD, Interstitial lung disease; IQR, interquartile ranges; NOS, Not otherwise specified; PCT, Pembrolizumab plus chemotherapy; PD-L1, programmed cell death protein 1; PSM, propensity score matching; SMD, Standardized mean difference.

### Efficacy

**Figure 1:**
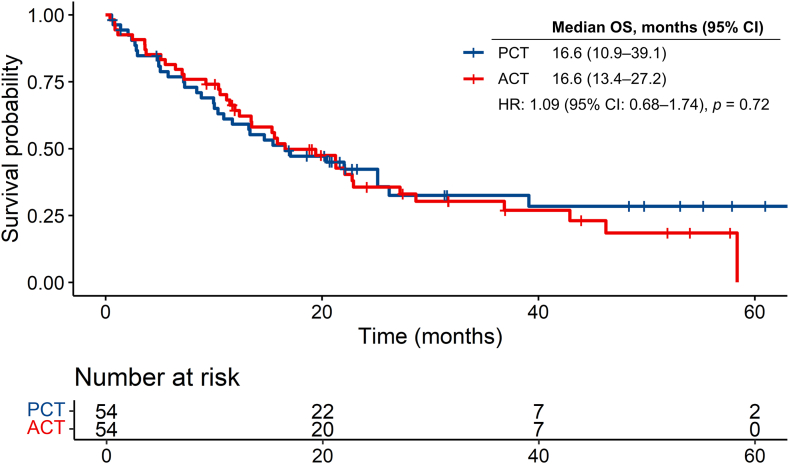
Kaplan-Meier OS curves of the PCT and ACT groups. ACT, atezolizumab plus chemotherapy; CI, confidence interval; HR, hazard ratio; OS, overall survival; PCT, pembrolizumab plus chemotherapy.

The median follow-up periods of the PCT and ACT groups were 15.1 months (interquartile range, 5.22–23.1) and 15.5 months (interquartile range, 9.28–26.4 mo), respectively (*p* = 0.59). The median ICI treatment cycles in the PCT and ACT groups were 7.0 (95% confidence interval [CI]: 2.25–12.5) and 5.5 (95% CI: 1.25–11.8), respectively (*p* = 0.25). OS events of 33 (61.1%) patients in the PCT group and 38 (70.4%) patients in the ACT group were confirmed. The median OS of the PCT group was 16.6 months (95% CI: 10.9–39.1 months), and that of the ACT group was 16.6 months (95% CI: 13.4–27.2 mo) (HR: 1.09; 95% CI: 0.68–1.74; *p* = 0.72) (Fig. 1).

The results of the subgroup analysis of OS for each age group are detailed in Supplementary Fig. 2. The median OS of patients 65 to 74 years of age in the PCT group was 16.6 months (95% CI: 8.83 mo–not reached [NR]); however, that of patients 65 to 74 years of age in the ACT group was 19.4 months (95% CI: 13.4–42.9 mo) (HR: 1.09; 95% CI: 0.61–1.96; *p* = 0.76) (Supplementary Fig. 2*A*). An analysis of patients 75 years or older indicated that the median OS of the PCT group was 17.1 months (95% CI: 11.7 mo–NR); however, that of patients 75 years or older in the ACT group was 15.6 months (95% CI: 11.5 mo–NR) (HR: 1.06; 95% CI: 0.48–2.33; *p* = 0.89) (Supplementary Fig. 2*B*).

Treatment responses to PCT and ACT after PSM are presented in Table 2. No significant differences in the Response Evaluation Criteria in Solid Tumors evaluation results were observed between groups. The objective response rate of both groups was 51.9%.

**Table 2:** Objective Response

Objective Response	PCT (n = 54)	ACT (n = 54)	*p* Value
RECIST (%)			0.10
CR	1 (1.9)	6 (11.1)	
PR	27 (50.0)	22 (40.7)	
SD	16 (29.6)	9 (16.7)	
PD	8 (14.8)	13 (24.1)	
NE	2 (3.7)	4 (7.4)	
ORR (%)	28 (51.9)	28 (51.9)	>0.99
DCR (%)	44 (81.5)	37 (68.5)	0.18

ACT, Atezolizumab plus chemotherapy; CR, Complete response; NE, Not evaluable; ORR, Objective response rate; PCT, Pembrolizumab plus chemotherapy; PD, Progressive disease; PR, Partial response; RECIST, Response Evaluation Criteria in Solid Tumors; SD, Stable disease.

### Safety

Treatment-related grade ≥3 AEs in each group before PSM are presented in Supplementary Table 1. Although the overall incidence of grade ≥3 AEs was not significantly different between groups (33.9% in the PCT group versus 35.4% in the ACT group; *p* = 0.95), the incidence of pneumonitis in the PCT group was significantly higher than that in the ACT group (29.4% versus 4.6%; *p* < 0.001). Treatment-related grade ≥3 AEs in each group after PSM are summarized in Table 3. The frequency of grade ≥3 AEs was not significantly different between groups (40.7% in the PCT group versus 33.3% in the ACT group; *p* = 0.55). These results were consistent with those of the age 65 to 74 years subgroup (40.0% in both groups; *p* >0.99) and the 75 years or older subgroup (36.8% in the PCT group versus 21.1% in the ACT group; *p* = 0.47).

**Table 3:** Grade 3 or Higher Adverse Events

Adverse Events	PCT (n = 54)	ACT(n = 54)	*P* Value
Severe AEs (%)	22 (40.7)	18 (33.3)	0.55
Pneumonitis	11 (20.3)	2 (3.7)	0.02
FN	4 (7.4)	2 (3.7)	0.68
Hepatobiliary toxicity	2 (3.7)	1 (1.9)	>0.99
Skin disorders	2 (3.7)	1 (1.9)	>0.99
Adrenal pituitary disorder	1 (1.9)	3 (5.5)	0.61
Gastrointestinal disorders	1 (1.9)	3 (5.5)	0.61
Neuromuscular disease	1 (1.9)	1 (1.9)	>0.99
Thrombocytopenia	0 (0.0)	2 (3.7)	0.50
Renal disorder	1 (1.9)	0 (0.0)	>0.99
Malaise	1 (1.9)	0 (0.0)	>0.99
Peripheral neuropathy	0 (0.0)	1 (1.9)	>0.99
Anaphylaxis	0 (0.0)	1 (1.9)	>0.99
Cardiac disorder	0 (0.0)	1 (1.9)	>0.99
Fever	0 (0.0)	1 (1.9)	>0.99

ACT, Atezolizumab plus chemotherapy; AEs, Adverse events; FN, Febrile neutropenia; PCT, Pembrolizumab plus chemotherapy.

**Figure 2:**
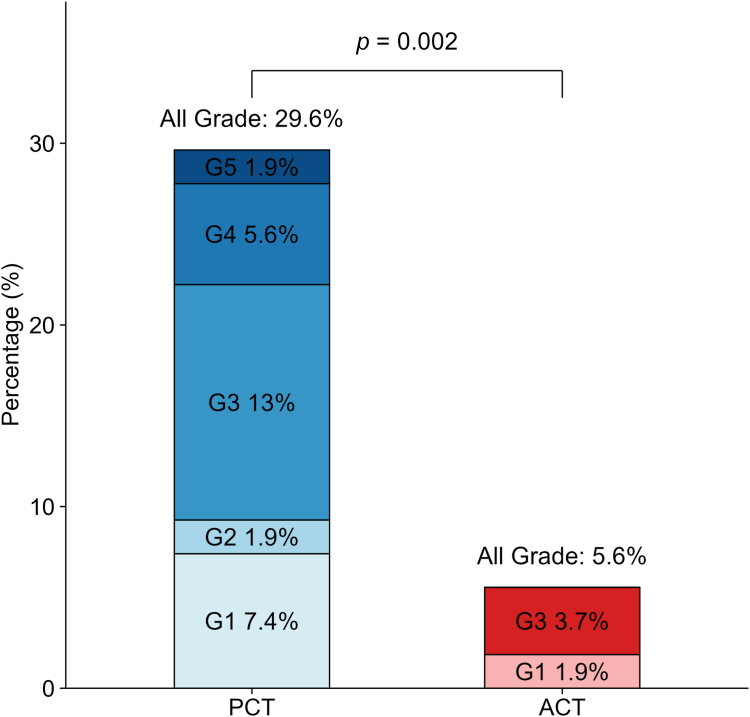
Patients in the PCT and ACT groups who developed treatment-related pneumonitis. ACT, atezolizumab plus chemotherapy; PCT, pembrolizumab plus chemotherapy.

Information regarding treatment-related pneumonitis after PSM is presented in Figure 2. The frequency of all-grades treatment-related pneumonitis of the PCT group (16 of 54 patients; 29.6%) was significantly (*p* = 0.002) higher than that of the ACT group (3 of 54 patients; 5.6%). Eleven patients in the PCT group developed grade ≥3 pneumonitis, including one case of grade 5 pneumonitis. One patient in the ACT group experienced grade 1 pneumonitis and two patients developed grade 3 pneumonitis. Among the 11 patients in the PCT group who developed grade ≥3 pneumonitis, two had preexisting ILD, including the one patient who developed grade 5 pneumonitis. In contrast, two patients in the ACT group who developed grade ≥3 pneumonitis did not have preexisting ILD. In the age 65 to 74 years subgroups of the PCT and ACT groups, pneumonitis was observed in 11 of 35 (31.4%) patients and 2 of 35 (5.7%) patients, respectively. In the 75 years or older subgroups of the PCT and ACT groups, pneumonitis was observed in four of 19 (21.1%) patients and one of 19 (5.3%) patients, respectively. In addition, in the ACT group, the pneumonitis rates of patients who received nab-PTX and those who received pemetrexed were similar (5.9% versus 5.0%; *p* > 0.99).

## Discussion

This real-world study compared the efficacy and safety of pembrolizumab and those of atezolizumab when combined with chemotherapy for patients 65 years or older with advanced or recurrent nonsquamous NSCLC. Although there was no statistically significant difference in OS between groups after PSM, ACT was associated with a lower risk of treatment-related pneumonitis. To the best of our knowledge, this is the first real-world study to compare ACT and PCT. These findings suggest that ACT offers better safety and could be a reasonable treatment option for older patients.

In the present study, the median OS of the ACT group was 16.6 months. The only study that previously provided data regarding ACT for older patients with NSCLC was the integrated analysis of the IMpower130 and IMpower132 trials; however, no such real-world study has been conducted.^12^ The integrated analysis revealed a median OS of 18.6 months for the overall population and a median OS of 28.2 months for the 75 years or older subgroup of the ACT group, which were much better than the OS observed during the present study.^12^ The difference in participant backgrounds could be attributable to the difference in prognoses between studies. For example, 5.6% of the patients in the present study had a performance status of 2 or greater, and 13% of the patients in the ACT group had preexisting ILD and were excluded from clinical trials. The present study provided prognostic information regarding ACT in real-world settings for the first time.

However, several studies of PCT for older patients in real-world settings have been published. A single-arm study reported that the OS of patients 75 years or older was significantly shorter than that of patients younger than 75 years when treated with PCT.^13^ This study further revealed that the OS of patients 75 years or older was shorter with the pemetrexed regimen, but that no significant difference was observed with the paclitaxel regimen. These results agree with those of the subgroup analyses of patients 75 years or older during clinical trials. According to the KEYNOTE-189 trial, the HR for OS of PCT compared with chemotherapy alone was 1.54 (95% CI: 0.76–3.14); however, it was 0.81 (95% CI: 0.43–1.55) according to the KEYNOTE-407 trial.^14^ These results suggest that the safety of the regimen comprising pembrolizumab and pemetrexed may be influenced by age. The aforementioned real-world study reported a median OS of 16.5 months for patients who received pembrolizumab with platinum and pemetrexed, which was comparable to that observed in our study.^13^ We only included patients with nonsquamous NSCLC who received pembrolizumab and platinum plus pemetrexed to allow comparisons with atezolizumab regimens. Histological diagnosis should be considered when determining, which treatment regimens to use for older patients.

The incidence of pneumonitis for patients in the PCT group in this study (29.6%) was much higher than that observed during the KEYNOTE-189 trial (4.4%).^10^ This discrepancy may be explained by the older age of the participants and high prevalence of preexisting ILD in our study because these factors are associated with a higher risk of immune-related pneumonitis.^15^, ^16^, ^17^, ^18^ In addition, because the Japanese subgroup in the KEYNOTE-189 trial developed pneumonitis at a higher rate than that of the overall population (8.0% versus 4.4%),^10^^,^^19^ and because a previous retrospective multicenter study in Japan that evaluated the efficacy and safety of the PCT regimen reported a higher incidence of pneumonitis (18.0% for all-grade and 5.0% for grade ≥3 pneumonitis),^20^ racial differences may have influenced this result. In the present study, although all participants were Japanese, and although the PCT and ACT groups had a comparable median age and similar prevalence of preexisting ILD, patients in the ACT group had a significantly lower risk of pneumonitis. Differences between groups may have been related to the mechanistic distinction between anti–PD-1 and PD-L1 antibodies. The incidence of pneumonitis with atezolizumab is lower than that with pembrolizumab when used as monotherapy.^21^ Compared with anti–programmed cell death protein 1 (PD-1) antibody, anti–PD-L1 antibody is associated with a lower risk of pneumonitis when used as monotherapy because of the difference in blockage coverage.^21^ Anti–PD-L1 antibody combines with PD-L1, thus, preventing its interaction with PD-1. In contrast, anti–PD-1 antibody binds to PD-1 and prevents its interaction with both PD-L1 and PD-L2.^21^ This binding range may lead to differences in immune activity and the incidence of immune-related AEs between anti–PD-L1 and anti–PD-1 antibodies, particularly when used in combination with chemotherapy. According to the results of the present study, when combined with chemotherapy, atezolizumab may be safer than pembrolizumab in terms of the development of pneumonitis.

The present study had several limitations. First, this study was a retrospective cohort study. Although PSM was used to adjust for background differences, selection bias may have existed for variables not included as covariates in the propensity score. Second, diagnoses of AEs were based on medical records; however, the incidence and severity of AEs were assessed by attending physicians and were not independently centrally reviewed. Third, the regimen of atezolizumab plus carboplatin plus pemetrexed is not approved in some countries. However, because this regimen was found to be effective for elderly patients by an integrated analysis of the IMpower130 and IMpower132 trials, and because it is a widely used regimen in Japan, we believe it was reasonable to include it in the ACT group. Fourth, although the incidence of pneumonitis among patients receiving pemetrexed and nab-paclitaxel within the ACT group was similar in the present study, differences in chemotherapy regimens between the PCT and ACT groups may have contributed to the overall difference in the pneumonitis incidence. Fifth, data regarding previous radiotherapy to the thorax and thoracic spine were not collected. Such treatment may have contributed to the development of treatment-related pneumonitis. Sixth, this study was conducted in Japan, and the cohort consisted of a single ethnic population.

In conclusion, this study found that compared with PCT, ACT provided similar survival and a lower risk of treatment-related pneumonitis when used for older Japanese patients in a real-world setting.

## CRediT Authorship Contribution Statement

**Kensuke Kanaoka:** Conceptualization, Data curation, Formal analysis, Investigation, Methodology, Resources, Visualization, Writing - original draft, Writing - review and editing.

**Kinnosuke Matsumoto:** Conceptualization, Data curation, Investigation, Methodology, Project administration, Resources, Supervision, Writing - review and editing.

**Takayuki Shiroyama:** Conceptualization, Data curation, Formal analysis, Investigation, Methodology, Project administration, Resources, Supervision, Visualization, Writing - review and editing.

**Akihiro Tsukaguchi:** Data curation, Investigation, Resources, Writing - review and editing.

**Koki Moritomo:** Data curation, Investigation, Resources, Writing - review and editing.

**Nao Shoshihara:** Data curation, Investigation, Resources, Writing - review and editing.

**Yuhei Kinehara:** Data curation, Investigation, Resources, Writing - review and editing.

**Yasuhiro Mihashi:** Data curation, Investigation, Resources, Writing - review and editing.

**Tomoki Kuge:** Data curation, Investigation, Resources, Writing - review and editing.

**Midori Yoneda:** Data curation, Investigation, Resources, Writing - review and editing.

**Soichiro Kato:** Data curation, Investigation, Resources, Writing - review and editing.

**Keijiro Yamauchi:** Data curation, Investigation, Resources, Writing - review and editing.

**Tomohiro Machiyama:** Data curation, Investigation, Resources, Writing - review and editing.

**Yuki Nishikawa:** Data curation, Investigation, Resources, Writing - review and editing.

**Osamu Morimura:** Data curation, Investigation, Resources, Writing - review and editing.

**Akito Miyazaki:** Data curation, Investigation, Resources, Writing - review and editing.

**Kiyohide Komuta:** Data curation, Investigation, Resources, Writing - review and editing.

**Kouji Azuma:** Data curation, Investigation, Resources, Writing - review and editing.

**Satoshi Tanaka:** Data curation, Investigation, Resources, Writing - review and editing.

**Toshie Niki:** Data curation, Investigation, Resources, Writing - review and editing.

**Akio Osa:** Data curation, Investigation, Resources, Writing - review and editing.

**Akihiro Tamiya:** Conceptualization, Data curation, Investigation, Project administration, Resources, Supervision, Writing - review and editing.

**Motohiro Tamiya:** Conceptualization, Data curation, Investigation, Project administration, Resources, Supervision, Writing - review and editing.

**Hidekazu Suzuki:** Conceptualization, Data curation, Investigation, Project administration, Resources, Supervision, Writing - review and editing.

**Masahide Mori:** Conceptualization, Data curation, Investigation, Project administration, Resources, Supervision, Writing - review and editing.

**Yoshito Takeda:** Project administration, Supervision, Writing - review and editing.

**Atsushi Kumanogoh:** Project administration, Supervision, Writing - review and editing.

## Disclosure

Dr. A. Tamiya reports receiving grants (from 10.13039/100004325AstraZeneca, 10.13039/100017239BeiGene, Taiho Pharmaceutical and Daiichi-Sankyo) and honoraria for lectures (from Amgen, AstraZeneca, Boehringer Ingelheim, Bristol-Myers Squibb, Chugai Pharmaceutical, DNA Chip Research, Eli Lilly, Kyowa-Kirin, Merck BioFarma, Merck Sharp & Dohme, Nihon-Kayaku, Novartis, Ono Pharmaceutical, Pfizer, Pulmonx, Taiho Pharmaceutical, Takeda Pharmaceutical, Thermo Fischer Scientific, and Tsumura) outside of the submitted work. Dr. Kinehara reports receiving honoraria for lectures (from AstraZeneca, Boehringer Ingelheim, Bristol-Myers Squibb, Chugai Pharmaceutical, Merck Sharp & Dohme, and Ono Pharmaceutical) outside of the submitted work. Dr. Matsumoto reports receiving grants (from 10.13039/100014422Eli Lilly Japan) and honoraria for lectures (from Bristol-Myers Squibb, Chugai Pharmaceutical, Eli Lilly Japan, Merck Sharp & Dohme, and Ono Pharmaceutical) outside of the submitted work. Dr. M. Tamiya reports receiving grants (from Boehringer Ingelheim, Bristol-Myers Squibb, and Ono Pharmaceutical) and honoraria for lectures (from Asahi Kasei Pharmaceutical, AstraZeneca, Boehringer Ingelheim, Bristol-Myers Squibb, Chugai Pharmaceutical, Eli Lilly, Merck Sharp & Dohme, Ono Pharmaceutical, and Taiho Pharmaceutical) outside of the submitted work. Dr. Mori reports receiving honoraria for lectures (from AbbVie, AstraZeneca, Boehringer Ingelheim, Chugai Pharmaceutical, Daiichi-Sankyo, Eli Lilly, Kyowa-kirin, MSD, Nihon-kayaku, Novartis, Ono Pharmaceutical, Phizer, Taiho, and Takeda) outside of the submitted work. Dr. Suzuki reports receiving honoraria for lectures (from AstraZeneca, Chugai Pharmaceutical, and Merck Sharp & Dohme) outside of the submitted work. The remaining authors declare no conflict of interest.

## Figures and Tables

**Supplementary Figure 2:**