# Supplementary material for: A Multicenter, Retrospective, Real-World Study of Atezolizumab Plus Chemotherapy and Pembrolizumab Plus Chemotherapy for Older Patients With NSCLC
Source: JTO Clin Res Rep. 2025 Aug 21;6(11):100891. doi: 10.1016/j.jtocrr.2025.100891 (PMC12547812; doi:10.1016/j.jtocrr.2025.100891)
Supplement: Supplementary Table 1 [file mmc1.docx]

|  | PCT (n=221) | ACT (n=65) | *p*-value |
| --- | --- | --- | --- |
| Pneumonitis (all grade) (%) | 65 (24.8) | 3 (4.6) | <0.001 |
| Pneumonitis (grade ≥3) (%) | 34 (15.3) | 2 (3.1) | 0.006 |
| Severe AE (%) | 75 (33.9) | 23 (35.4) | 0.95 |
| FN | 8 (3.6) | 4 (6.2) | 0.48 |
| Hepatobiliary toxicity | 8 (3.6) | 1 (1.5) | 0.69 |
| Skin disorders | 8 (3.6) | 1 (1.5) | 0.69 |
| Adrenal pituitary disorder | 4 (1.8) | 3 (4.6) | 0.20 |
| Renal disorder | 4 (1.8) | 0 (0.0) | 0.58 |
| Gastrointestinal disorders | 3 (1.4) | 4 (6.2) | 0.049 |
| Neuromuscular disease | 3 (1.4) | 1 (1.5) | >0.99 |
| Neutropenia | 3 (1.4) | 0 (0.0) | >0.99 |
| Thrombocytopenia | 0 (0.0) | 2 (3.1) | 0.051 |
| Anaphylaxis | 0 (0.0) | 2 (3.1) | 0.051 |
| Anemia | 1 (0.5) | 0 (0.0) | >0.99 |
| Arthritis | 1 (0.5) | 0 (0.0) | >0.99 |
| Bacterial pneumonia | 1 (0.5) | 0 (0.0) | >0.99 |
| CPK elevation | 1 (0.5) | 0 (0.0) | >0.99 |
| Malaise | 1 (0.5) | 0 (0.0) | >0.99 |
| Myocarditis | 1 (0.5) | 0 (0.0) | >0.99 |
| Pancytopenia | 1 (0.5) | 0 (0.0) | >0.99 |
| Pulmonary embolism | 1 (0.5) | 0 (0.0) | >0.99 |
| Cardiac disorder | 0 (0.0) | 1 (1.5) | 0.23 |
| Fever | 0 (0.0) | 1 (1.5) | 0.23 |
| Hemophagocytic syndrome | 0 (0.0) | 1 (1.5) | 0.23 |
| Pancreatitis | 0 (0.0) | 1 (1.5) | 0.23 |
| Peripheral neuropathy | 0 (0.0) | 1 (1.5) | 0.23 |
| Thyroid disorder | 0 (0.0) | 1 (1.5) | 0.23 |

Supplementary Table 1 Adverse events before propensity score matching
